# The role of self-efficacy and control beliefs in response to a multimodal headache intervention: results from a prospective observational study with a waiting-list comparator

**DOI:** 10.1038/s41598-026-47295-y

**Published:** 2026-04-14

**Authors:** Luise Bartsch, Nadja Fiebig, Christine Klötzer, Sebastian Strauß, Uwe Reuter, Robert Fleischmann

**Affiliations:** 1 Department of Neurology, University Medicine Greifswald, Ferdinand-Sauerbruch-Str. 1, 17475 Greifswald, Germany; 2 Department of Neurology, Charité - University Medicine Berlin, Berlin, Germany; 3 University Hospital Bonn, Bonn, Germany

**Keywords:** Migraine, Headache, Multimodal treatment, Predictor, Self-efficacy, Locus of control, Psychotherapy, Behavioral therapy, Diseases, Health care, Medical research, Neurology, Neuroscience

## Abstract

Primary headache disorders, particularly migraine, rank among the leading causes of disability worldwide and impose substantial social and economic burden. Multimodal headache treatment (MMHT) combines pharmacologic, physiotherapeutic and cognitive-behavioural interventions and has shown benefit in high-burden patients, yet the psychological factors associated with sustained improvement remain unclear. Self-efficacy (SE) supports active coping, whereas the chance-related multidimensional health locus of control (CMHLC-C) reflects belief in uncontrollable outcomes. We hypothesised that these control beliefs are modifiable through intervention and that their baseline levels are associated with subsequent improvement in headache burden. Adults with primary headache disorders were enrolled in a prospective observational study and participated in a one-week MMHT at a tertiary neurological day clinic. Headache impact (HIT-6; primary endpoint), monthly headache days (MHD), and highest headache pain severity (HHPS) were measured three months before treatment (waiting-list baseline), at the start and end of MMHT, and at 3-, 6-, and 9-month follow-up. The waiting-list period served as a within-person pre-treatment comparator for spontaneous change. Baseline headache-management SE and MHLC-C subscales were tested as predictors of longitudinal HIT-6 trajectories using a generalized linear mixed model with visit as a repeated factor. Sixty-five patients were included in the analytic cohort (84.6% female; mean age 40.6 ± 13.3 years; 89.8% migraine). HIT-6 decreased from 63.2 at the waiting-list baseline to 59.0 at nine months (*p*<.001). MHD was reduced from 17.9 ± 8.0 to 12.2 ± 7.5 days, and 44% experienced a ≥ 30% reduction. HHPS also decreased throughout follow-up. Mean SE increased significantly during MMHT and early follow-up, whereas CMHLC-C showed only small changes over time. The mixed model was significant (F(2,165) = 7.25, *p*<.001). Higher baseline SE predicted larger reductions in HIT-6 (β=−0.55, t(165) = − 2.62, *p*=.010), whereas greater belief in chance predicted smaller reductions (β = 0.85, t(165) = 2.80, *p*=.006). In this prospective observational study with a waiting-list comparator, MMHT was associated with sustained reductions in headache impact, headache frequency, and pain severity. Baseline self-efficacy and chance-related control beliefs independently predicted treatment response and may represent clinically relevant stratification targets in personalised headache care.

## Introduction

Primary headache, such as migraine or tension-type headache are amongst the most common neurological disorders and a leading cause of disability worldwide^1^. With a one-year prevalence of 15%, Migraine is the most debilitating neurological disease^2^. In Germany, 14.8% of women and 6.0% of men suffer from migraine, although it is assumed that the number of unreported cases is higher^3^. In addition to the primary symptom of headache, individuals with migraine suffer from a wide range of debilitating comorbidities, including mood disturbances, psychosocial difficulties, reduced life satisfaction, and a higher likelihood of developing psychiatric disorders such as depression and anxiety^4–7^. This complex array of symptoms significantly impacts the quality of life for those affected and often leads to chronicity, further complicating treatment^8^. Given the biopsychosocial model of migraine, effective, sustainable treatment strategies are crucial. Research suggests that multidisciplinary, multimodal approaches can improve outcomes in high-burden headache patients; however, evidence for day-clinic programs is largely based on observational cohorts with heterogeneous follow-up, and controlled long-term data remain limited^9,10^. In addition to reducing the burden on healthcare systems, such interventions aim to address the substantial limitations migraines impose on daily life, making sustainable improvements a key goal.

Multidisciplinary headache treatment programs (MMHT) that combine pharmacological and non-pharmacological elements have shown promise in treating chronic pain conditions like migraine. These programs aim to provide patients with tools to manage their disease through a biopsychosocial approach. Such programs typically include medical care, psychological support, and physical therapy, emphasizing techniques like relaxation, progressive muscle relaxation, and mindfulness, which patients can integrate into their everyday routines. However, while some studies have demonstrated the short-term efficacy of such interventions, questions remain about their long-term benefits and the mechanisms responsible for sustained improvements^10–12^. Given the chronic nature of migraine and its significant burden on individuals and the healthcare system, it is critical to determine whether short-term, intensive interventions can produce lasting benefits and which factors contribute to these outcomes.

This study focuses on evaluating whether MMHT leads to sustainable reductions in the daily life impairments caused by migraine, as well as decreases in headache frequency and intensity. Given the chronic nature of migraine and its substantial burden on individuals and the healthcare system, it is important to determine whether short, intensive interventions can produce lasting benefits and which patient-related factors are associated with these outcomes. The present study therefore examined whether MMHT is associated with sustained reductions in headache-related impairment, headache frequency, and pain severity in a tertiary day-clinic setting. In addition, we investigated whether baseline headache-specific self-efficacy and chance-related control beliefs predict longitudinal improvement in headache impact. Self-efficacy, defined as the belief in one’s ability to influence symptoms and coping, has been associated with better outcomes in chronic pain and headache management, whereas externalised control beliefs may be linked to less adaptive coping. By focusing on these constructs, the study aims to identify psychologically meaningful predictors of response to multimodal headache care.

## Methods

### Study design and participants

This study employed a prospective observational design with a within-person waiting-list comparator to evaluate a one-week multimodal day-clinic intervention for patients with primary headache disorders. Patients presenting to the headache clinic who met diagnostic criteria for a primary headache disorder according to the International Classification of Headache Disorders (ICHD-3) were eligible for inclusion^13^. Participants were 18 years or older, exhibited either impending or established chronicity of their headache disorder, and had not achieved sufficient improvement with prior unimodal therapies. All participants provided written informed consent, and the study was approved by the local ethics committee (BB 004/21).

Participants entered the study either through internal waiting (patients already listed for MMHT within the clinic) or external waiting (patients referred from outside institutions awaiting treatment). Both groups underwent a waiting period of approximately three months before beginning MMHT. During this waiting-list phase, no additional structured day-clinic intervention was provided; however, usual care continued, including acute and preventive treatments as clinically indicated. This pre-treatment interval served as a within-person comparator for short-term spontaneous change before therapy onset.

Study assessments were conducted at six time points: at study entry (waiting-list baseline, approximately three months before MMHT), at the start and end of MMHT, and at 3-, 6-, and 9-month follow-up. The comparison between the waiting-list baseline and the start of MMHT captured pre-treatment variation, whereas subsequent changes were interpreted as post-intervention trajectories within the limits of an observational design.

### Definition of self-efficacy and locus of control

Self-efficacy and health-related control beliefs are established psychological constructs in chronic pain and headache research. In the context of headache disorders, self-efficacy refers to the belief that one can actively influence symptom management, cope with attacks, and maintain functioning despite burden. Locus of control refers to whether health outcomes are perceived as primarily determined by one’s own actions or by external factors such as chance. Prior headache research suggests that higher self-efficacy and more internalised control beliefs are associated with more adaptive coping and better outcomes, whereas stronger external or chance-related beliefs may be linked to less active headache management^14^. These constructs were therefore selected as psychologically meaningful predictors of response to multimodal treatment (Table 1).

### Multimodal headache treatment

The MMHT was implemented in a one-week day-clinic format at the Neurological Day Clinic of a tertiary headache center in northern Germany. The program combined medical consultations and individualized management planning with occupational therapy, physiotherapy/exercise therapy, relaxation techniques, biofeedback where indicated and available, and both group and individual psychological interventions. The psychological component comprised five group sessions focusing on relaxation techniques, mindfulness-based elements, and CBT-informed headache management strategies, including trigger/stress management, coping skills, and self-management. Patients additionally received individual psychological counselling addressing patient-specific coping strategies, headache education, and behavioural implementation in daily life. The non-pharmacological components were designed to teach practical skills transferable to everyday routines. To enhance reproducibility, the structure, disciplines involved, and approximate dose of all treatment modules are summarized in Table 2^15^.

**Table 1:** Baseline characteristics of the study sample.

Category	Variable	Baseline descriptives
	Age, years	40.63 (13.25)
	Monthly headache days (MHD)	17.87 (8.40)
	Headache intensity (HHPS)	6.60 (1.90)
Sex	Female	55 (84.6%)
	Male	10 (15.4%)
Headache frequency category*	< 15 headache days/month	19 (29.2%)
	≥ 15 headache days/month	46 (70.8%)
Diagnosis (ICHD-3)	Migraine (any)	59 (89.8%)
	- Migraine without aura	25 (38.5%)
	- Migraine with aura	32 (49.2%)
	- Hemiplegic migraine	2 (3.1%)
	Tension-type headache	4 (6.1%)
	Cluster headache	1 (1.5%)
Referral source	Internal waiting-list	46 (70.8%)
	External waiting-list	19 (29.2%)

The cohort is representative of a clinically severely affected tertiary-care headache population, consisting predominantly of patients with migraine and characterized by both high headache frequency and marked symptom severity. Continuous data are presented as mean ± standard deviation; categorical data are presented as n (%).

**Table 2:** Core components of the 5-day multimodal headache treatment (MMHT) day-clinic program The MMHT is delivered as a structured, interdisciplinary day-clinic program over five consecutive weekdays.

Domain/module	Format & dose	Delivered by	Core content (examples)	Transfer / homework
Neurological headache assessment & education	Small-group teaching + individual review; daily brief check-ins	Neurologist / headache specialist nurse	ICHD-3 diagnosis review; red-flag education; acute vs. preventive strategy; medication-overuse prevention; individualized treatment plan	Personalized action plan; headache diary instructions
Physiotherapy / exercise therapy	Daily session (group) + individualized exercises	Physiotherapist	Neck/shoulder assessment; posture/ergonomics; graded activity; stretching/strengthening; endurance planning	Home exercise plan; activity pacing
Relaxation training	Daily guided practice	Psychologist / trained therapist	Progressive muscle relaxation, breathing techniques, mindfulness-based exercises	Audio-guided practice; daily relaxation routine
Biofeedback (as indicated)	1–2 sessions per week; individualized	Psychologist / biofeedback therapist	Physiological self-regulation (e.g., EMG/thermal) depending on availability and indication	Skill practice between sessions
Psychological individual and group intervention (CBT-informed)	Several individual and group sessions across the week	Psychologist	Trigger/stress management; cognitive restructuring; coping with fear of attacks; sleep hygiene; goal setting; self-efficacy building	Worksheet-based practice; relapse prevention plan
Occupational therapy / daily-life skills (optional/indicated)	Group or individual session(s)	Occupational therapist	Workplace/home adaptations; energy management; sensory modulation strategies	Implementation checklist
Nursing counseling / self-management	Brief daily check-ins + 1 structured session	Headache nurse	Medication reconciliation; practical self-management; adherence support; planning follow-up care	Self-monitoring and adherence prompts

Exact timing may vary slightly depending on group size and individual needs.

### Data collection and study design

Data were collected at six time points: waiting-list baseline (− 3 months), baseline at MMHT start (Day 1), end of MMHT (Day 5), and follow-up at 3, 6, and 9 months. The waiting-list baseline provided a pre-treatment reference for short-term symptom stability and spontaneous change. Day 1 served as the baseline for evaluating post-intervention trajectories after the start of MMHT.

### Questionnaires

The primary outcome measure for the MMHT efficacy for improving headache-related quality of life was the Headache Impact Test-6 (HIT-6), a well-validated tool for assessing the impact of headaches on daily life over the past month. HIT-6 scores range from 36 to 78, with higher scores indicating greater impairment. Scores were categorized into four levels: little or no impact (< 50), some impact (50–55), substantial impact (56–59), and very severe impact (≥ 60)^16,17^. Secondary outcomes included headache frequency, measured as the monthly headache days (MHD), and Highest Headache Pain Severity (HHPS), assessed using a numeric rating scale (NRS). The short form of the *FKMS-G-SF* is the German adaptation of the Headache Management Self-Efficacy Scale^18^. This questionnaire measures headache-specific self-efficacy, i.e. patients’ confidence in managing their headaches and their belief in their ability to control their headache symptoms. This questionnaire comprises 6 items and the response options are presented on a 7-point Likert scale^18^. The Multidimensional Health Locus of Control (MHLC-C) is a questionnaire for the detailed measurement of health-related locus of control beliefs^19^. Health locus of control refers to how much control a person perceive they have over their health behaviour. This instrument captures internal and external locus of control beliefs using the subscales “chance” (CMHLC-C), “other people” (OMHLC-C), “doctors” (DMHLC-C) and “internal” (IMHLC-C). For use in a German headache centre, we translated the questionnaire into German^20^. For economic and organizational reasons, the questionnaire was only used up to the 3-month follow-up.

### Statistical analysis

All analyses were performed using SPSS (v29.0). Analyses were conducted in a modified intention-to-treat (mITT) set including all participants with baseline data who attended at least one MMHT day (Day 1; *n* = 65). Participants who withdrew prior to Day 1 did not contribute baseline or outcome data and were therefore not included in the analytic dataset, but they are fully accounted for in the participant flow diagram. For longitudinal predictor analyses of HIT-6 trajectories, we used a generalized linear mixed model with visit as a repeated factor; this likelihood-based approach uses all available observations and can accommodate missing follow-up under a missing-at-random assumption. For non-parametric within-subject comparisons across time points, analyses were performed on available cases and denominators are reported The resulting z-values represent the standardized test statistics, while effect sizes (r) were derived as r = z / √N (N = sample size), interpreted as small≈0.1, medium≈0.3, and large≈0.5. For the predictor analysis, a generalized linear mixed model (GLMM) with repeated measures was fitted (normal distribution, identity link). Patient ID was specified as a random intercept and visits (waiting‑list baseline, start of MMHT, end of MMHT, and 3‑/6‑/9‑month follow‑ups) were treated as repeated measures with a diagonal covariance structure. Candidate baseline predictors included headache‑management self‑efficacy and the internal, chance and physician subscales of the MHLC‑C. Stepwise backward elimination removed non‑significant predictors until only variables with *p* < .05 remained. Model fit was evaluated with information criteria (AIC). Final parameter estimates were reported as regression coefficients with standard errors and 95% confidence intervals.

## Results

### Participants subject characteristics

**Fig. 1:**
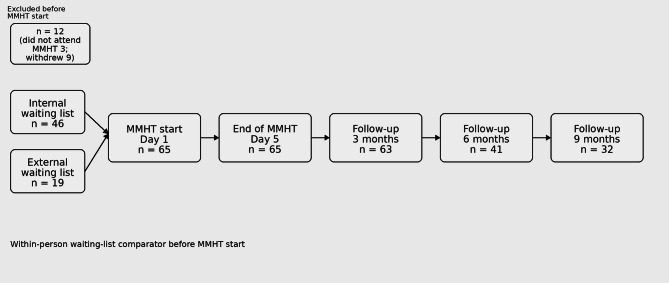
, Patient flow and study design. The flow diagram shows recruitment (*n* = 77), exclusions before MMHT start (*n* = 12; prior MMHT *n* = 3; withdrew before start *n* = 9), and the analytic cohort with baseline data (*n* = 65). All included participants completed a 3-month waiting-list phase with an assessment at − 3 months, followed by assessments at MMHT start (Day 1), end of MMHT (Day 5), and follow-up at 3, 6, and 9 months.

A total of 77 patients participated in the MMHT between January 2022 and April 2023. Of these, 12 patients were excluded (prior MMHT participation *n* = 3; withdrew before treatment start *n* = 9). As a result, 65 patients were included in the analytic cohort (Fig. 1). Of these, 55 were female (84.6%) and the mean age was 40.63 ± 13.25 years. Most participants were diagnosed with migraine (59/65, 89.8%), whereas other primary headache diagnoses were uncommon (tension-type headache *n* = 4; cluster headache *n* = 1). Baseline headache burden was high (MHD 17.87 ± 8.40), and 46/65 (70.8%) reported ≥ 15 headache days per month, indicating a high-frequency/chronic-range headache burden. Participants were recruited from both the University Medical Center’s outpatient clinic and external referrals.

### Efficacy of multimodal treatment for headache outcomes

**Fig. 2:**
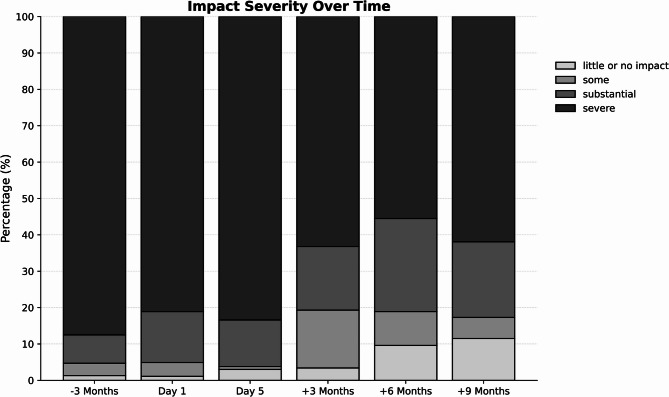
Changes in monthly headache days (MHD) and highest headache pain severity (HHPS) across the waiting-list phase and follow-up. Values are shown for the waiting-list baseline (− 3 months), baseline at MMHT start (Day 1), end of MMHT (Day 5), and follow-up at 3, 6, and 9 months. The waiting-list baseline is included to illustrate the pre-treatment trend (− 3 months to Day 1) relative to the post-MMHT trajectory. Data are presented as mean ± standard error.

Global tests for mean differences (Bonferroni-corrected) revealed significant main effects for both HIT-6 and MHD (each *p*<.001). At baseline, 70.8% of patients reported > 15 headache days per month. MHD decreased from 17.8 ± 8.0 at baseline to 12.2 ± 7.5 at 9-month follow-up, corresponding to a 32.5% reduction. Reductions were significant at nearly all time points: baseline (z = − 4.87, *p*<.001, *r*=.25), 3 months (z = − 8.97, *p*<.001, *r*=.46), 6 months (z = − 11.89, *p*<.001, *r*=.63) and 9 months (z = − 12.61, *p*<.001, *r*=.67), but not immediately after MMHT (z = − 1.16, *p*=.25, *r*=.06) versus waiting-list control (WLC). A ≥ 30% reduction in MHD was achieved by 44% of participants (Fig. 2).

HHPS declined from 6.6 ± 1.9 at baseline and showed significant reductions across all post-treatment assessments: after MMHT (z = − 5.84, *p*<0.001, *r*=.31), 3 months (z = − 6.12, *p*<0.001, *r*=0.32), 6 months (z = − 6.90, *p*<0.001, *r*=0.37) and 9 months (z = − 5.51, *p*<.001, *r*=.28) compared with WLC.

**Fig. 3:**
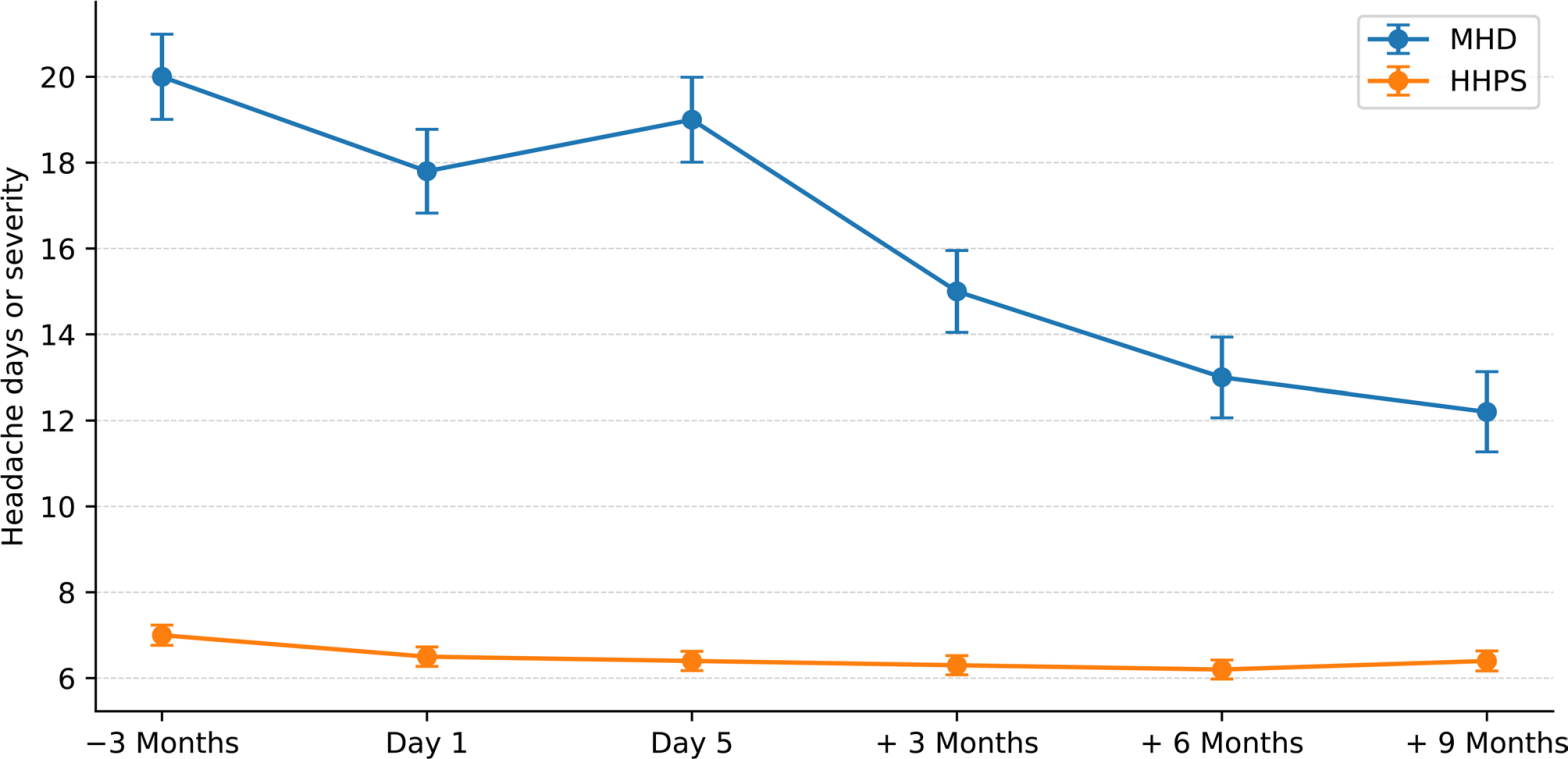
Distribution of HIT-6 impact severity categories across the waiting-list phase and follow-up. The distribution of HIT-6 severity categories is shown at the waiting-list baseline (− 3 months), baseline at MMHT start (Day 1), end of MMHT (Day 5), and follow-up at 3, 6, and 9 months. The waiting-list baseline is included to illustrate the pre-treatment trend relative to the post-MMHT trajectory.

For HIT-6, significant reductions were observed at baseline (z = − 6.37, *p*<0.001, *r*=.32), 3 months (z = − 9.90, *p*<0.001, *r*=0.51), 6 months (z = − 13.25, *p*<0.001, *r*=0.51) and 9 months (z = − 11.20, *p*<.001, *r*=0.71), but not at the end of MMHT (z = − 1.40, *p*=0.16, *r*=0.07). Mean HIT-6 decreased from 63.0 at baseline to 59.0 at 9 months, with 37% of patients achieving a clinically meaningful improvement (≥ 5-point reduction; Fig. 3).

### Predictors of multimodal treatment efficacy

**Fig. 4:**
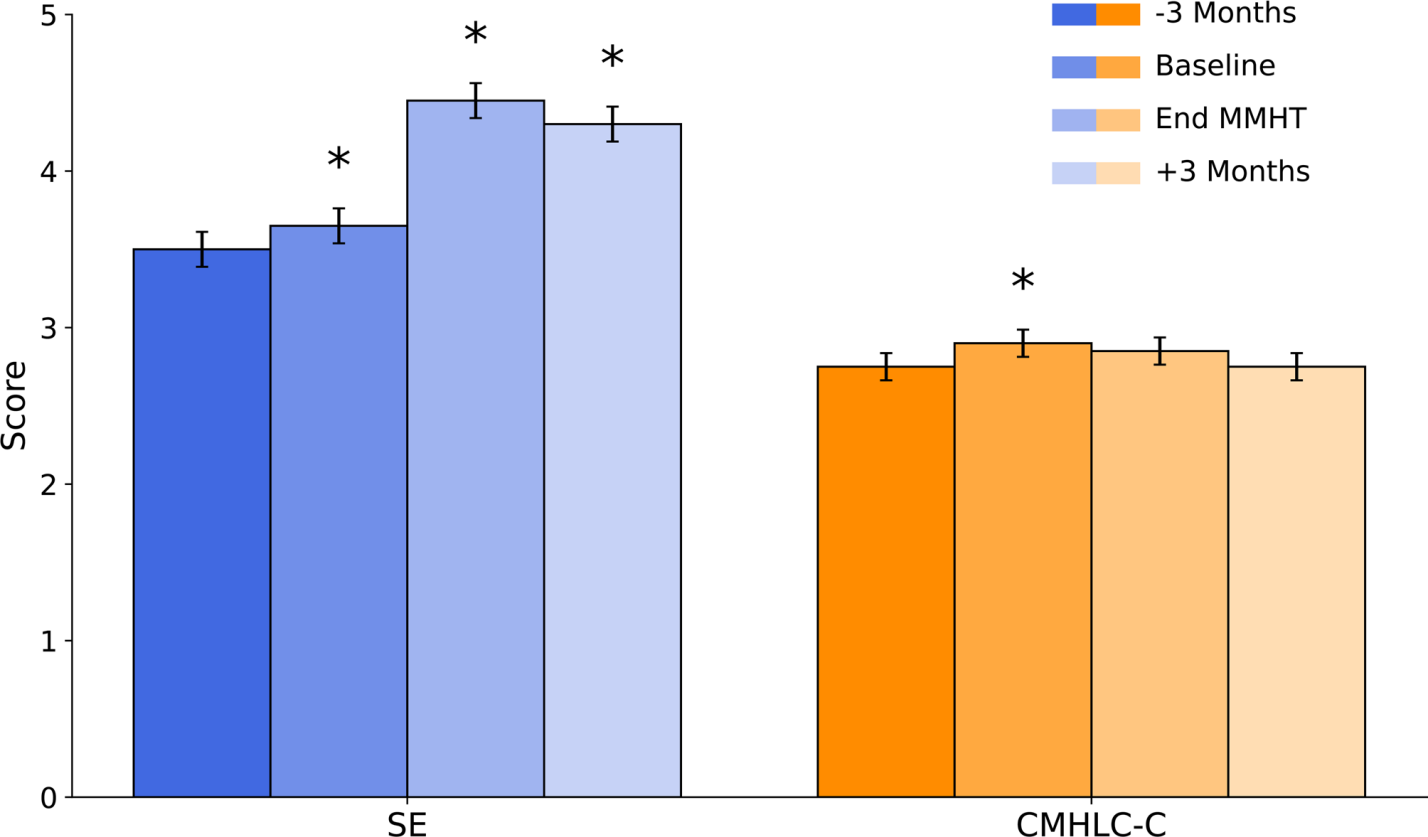
Changes in headache-management self-efficacy (SE) and chance-related locus of control (CMHLC-C) across the waiting-list phase and early follow-up. Values are shown at the waiting-list baseline (− 3 months), baseline at MMHT start (Day 1), end of MMHT (Day 5), and 3-month follow-up. Data are presented as mean ± standard error. Asterisks indicate statistically significant differences from the waiting-list baseline for SE; CMHLC-C showed only small, non-significant changes over time. MHLC-C subscales were assessed up to the 3-month follow-up to capture early psychological change proximal to the intervention.

There was a significant increase in SE, whereas CMHLC-C showed only a small, non-significant downward change over time (Fig. 4). Yet, their assessment as predictors of MMHT treatment effects was more relevant for the current study than group-level changes. GLMM including baseline predictors explained a significant proportion of the variance in HIT‑6 (F = 7.25(2,165), *p*<.001). In this model, higher baseline self‑efficacy was associated with lower HIT‑6 scores (β=−0.552, SE = 0.210, t(165) = − 2.623, *p*=.010), and the chance subscale of the MHLC‑C was associated with higher HIT‑6 scores (β = 0.851, SE = 0.305, t(165) = 2.795, *p*=.006). The intercept (63.2 ± 1.3) corresponds to the mean HIT‑6 score at the waiting‑list baseline. Internal and physician‑related locus‑of‑control subscales did not improve model fit and were excluded. Correlations among the predictors were modest (*r* ≤ .69), indicating acceptable multicollinearity. These results show that patients with greater confidence in their ability to manage headaches experienced larger reductions in headache impact across the follow‑up period, whereas patients attributing their health to chance showed smaller improvements.

## Discussion

In this prospective cohort, participation in a standardized 5-day interdisciplinary MMHT was associated with sustained improvements in headache burden and impact. Comparable multimodal headache programs in tertiary centers have reported clinically meaningful reductions in headache frequency and disability, supporting the concept of integrated headache care^10^. Our program combined individualized medical management with behavioural components, including CBT-informed strategies and, where indicated, biofeedback. Behavioural interventions such as CBT and biofeedback have demonstrated efficacy in migraine and may contribute to improvements in self-management and disability outcomes^15^.

A one-week MMHT may be sufficient to learn and practise lifestyle changes and to fill gaps in knowledge about drug and non-medication treatment. Other studies have found that only 37.9% of participants were aware of methods of attack relief^21^. There was also a significant reduction in headache disability and after 6- and 9-month follow-up to baseline. With regard to the HIT-6 score, there was a slight tendency towards an increase after six months. This suggests that a refresher program should be carried out after this period in order to maintain self-efficacy in the longer term. For example, an evaluation of the implementation of drug and non-drug treatment can be carried out in order to maintain the long-term effect of MMHT.

Participants in this cohort had a high baseline headache burden, with many reporting ≥ 15 headache days per month, a group that is often difficult to treat and frequently requires combined pharmacological and behavioural approaches. Behavioural mechanisms such as headache-management self-efficacy and control beliefs are considered relevant targets of psychological migraine treatments, and prior work has shown that behavioural migraine management can produce durable increases in self-efficacy and more internalised control beliefs^14^. In our study, self-efficacy increased during MMHT and early follow-up, and higher baseline self-efficacy predicted greater reductions in headache impact, whereas stronger chance-related control beliefs predicted smaller improvements. These findings support the relevance of expectancy-related mechanisms and suggest that multimodal programs may be particularly useful when they strengthen patients’ self-management resources.

### Limitations

This study has several limitations. First, it used a prospective observational design with a within-person waiting-list comparator rather than randomization to an attention-matched control. The waiting-list phase helps to characterize short-term spontaneous change before treatment onset, but it does not constitute a placebo condition and cannot disentangle specific intervention effects from contextual and expectancy-related influences. This is particularly relevant in migraine, where placebo responses are substantial and have increased over time across prevention trials^22^. Second, symptom fluctuation and regression to the mean may contribute to apparent improvement in repeated-measures designs^23^. Third, usual care continued during the waiting-list phase, and concomitant treatment changes during follow-up were not fully standardized, which may also have influenced outcomes. Finally, attrition over follow-up may have introduced bias, and the single-center tertiary setting may limit generalisability to other care contexts. Accordingly, the findings should be interpreted as evidence of association in a real-world interdisciplinary care setting rather than as proof of specific causal treatment effects.

## Data Availability

The datasets generated and/or analyzed during the current study are not publicly available due to legal restrictions on data sharing under the EU General Data Protection Regulation (GDPR; Regulation (EU) 2016/679), but are available from the corresponding author on reasonable request.
